# Potential of *Pleurotus ostreatus* Mycelium for Selenium Absorption

**DOI:** 10.1155/2014/681834

**Published:** 2014-06-04

**Authors:** Ivan Milovanović, Ilija Brčeski, Mirjana Stajić, Aleksandra Korać, Jelena Vukojević, Aleksandar Knežević

**Affiliations:** ^1^Faculty of Biology, University of Belgrade, Takovska 43, 11000 Belgrade, Serbia; ^2^Faculty of Chemistry, University of Belgrade, Studentski Trg 3, 11000 Belgrade, Serbia; ^3^Faculty of Biology, University of Belgrade, Center for Electron Microscopy, Studentski Trg 3, 11000 Belgrade, Serbia

## Abstract

The aim of this study was to evaluate the effect of high selenium (Se) concentrations on morphophysiological and ultrastructural properties of *Pleurotus ostreatus*. Mycelium growth was good in media enriched with 5.0, 10.0, and 20.0 mg L^−1^ of Se, concentration of 500.0 mg L^−1^ strongly inhibited growth, and 1000.0 mg L^−1^ was the minimum inhibitory concentration. Contrary to thin-walled, hyaline, branched, and anastomized hyphae with clamp-connections in the control, at Se concentrations of 100.0 and 500.0 mg L^−1^, they were noticeably short, frequently septed and branched, with a more intensive extracellular matrix, and without clamp-connections. At high Se concentrations, hyphae with intact membrane, without cellular contents, with a high level of vacuolization, and with numerous proteinaceous bodies were observed. Biomass yield ranged between 11.8 g L^−1^, in the control, and 6.8 g L^−1^, at an Se concentration of 100.0 mg L^−1^, while no production was detected at a concentration of 500.0 mg L^−1^. Se content in the mycelia reached a peak (938.9 **μ**g g^−1^) after cultivation in the medium enriched with Se at the concentration of 20.0 mg L^−1^, while the highest absorption level (53.25%) was found in the medium enriched with 5.0 mg L^−1^ Se.

## 1. Introduction

Recent rapid and progressive development of technology and industry has led to increasing the proportion of various environmental pollutants, such as pesticides, toxic xenobiotics, metals, metalloids, and halogenated and polycyclic aromatic hydrocarbons [1]. Nowadays, one of the frequent metalloid and potential air, water, and soil pollutants, which is widely used in various industries in the production of glass, pigments, pesticides, stainless steel, photoelectric cells, and cosmetics preparations due to distinctive physical and especially electrical properties, is Se [2]. Numerous human activities (mining, coal and oil usage, agricultural fertilization and irrigation, etc.) cause significant Se emission (37.5%–40.6% of the total Se emissions to the atmosphere) and ecosystem contamination with this trace element [2–4]. Thus, Se concentration in soil is mainly low (<1 ppm), but in seleniferous areas the amount could be significantly higher leading to its accumulation in plants and other organisms from the food chain and appearance of numerous disorders and diseases [5, 6]. However, total Se content is not an adequate indicator of the Se hazard, as the toxicity of Se compounds depends on their speciation and bioaccumulation. For instance, the lethal doses (LD_50_) can differ even 2680-fold between water-soluble selenite and insoluble elemental Se [2].

Selenium in trace concentrations is an essential microelement for normal human and animal growth and development due to its presence in a number of important enzymes and proteins [7–9]. However, at slightly higher concentrations, it becomes toxic and harmful because as a sulphur analogue it has the ability to be incorporated into sulphur-containing amino acids and proteins, changing their conformation and activity [10, 11].

Some edible and medicinal mushroom species accumulate Se at elevated concentrations, participating in its cycling in the environment [12, 13].* P. florida* fruiting bodies cultivated on wheat straw from the seleniferous site were highly enriched in Se [14], while data from polluted sites due to human activities are lacking [15]. However, numerous studies have demonstrated different capacities of Se absorption in various fungal species and even strains [16, 17]. Thus, Se content in* Boletus edulis *fruiting bodies could be up to 17.0 mg kg^−1^ of dry weight, in* B. luridus *21.5 mg kg^−1^, in* Macrolepiota *spp. 5.0 mg kg^−1^, in wild* Agaricus* spp. 2.7 mg kg^−1^, in* Lactarius torminosus* 1.9 mg kg^−1^, in* Lepista luscina* 2.5 mg kg^−1^, and in* Marasmius oreades* up to 1.6 mg kg^−1^ [18, 19]. Previous studies demonstrated that numerous species of the genus* Pleurotus* possess the ability to absorb Se from the medium [14, 20, 21]. Additionally, these authors demonstrated the high potential of* P. ostreatus* to absorb Se from a substrate enriched with the trace element to concentrations which ranged from 0.3 to 1.3 mg mL^−1^ amongst two varieties and 10 strains. However, tests on extremely high concentrations have never been done. Regarding the current findings that* Pleurotus* species are good producers of a large biomass pool and effective absorbers of metalloids and metals with high viability in their presence, they could have an important role in reducing the content and toxicity of the pollutant in the environment [22]. Defining the highest tolerant Se dose for mycelial growth of* P. ostreatus* as well as the effect of Se on morphophysiological features is of fundamental importance for further studies to assess their potential usage.

The purpose of this study was to resolve the question of how high Se concentrations affect biomass production, hyphae morphology, and ultrastructure, as well as the capacity of Se absorption and accumulation in* Pleurotus ostreatus*.

## 2. Materials and Methods

### 2.1. Organism and Cultivation Conditions

The culture of* P. ostreatus* HAI 592 originated from the Institute of Evolution, University of Haifa, Israel (HAI). It is preserved on malt agar (MA) medium in the culture collection of the Institute of Botany, Faculty of Biology, University of Belgrade.

The inoculum preparation involved the following steps: (i) inoculation of 100.0 mL of synthetic medium (glucose, 10.0 g L^−1^; NH_4_NO_3_, 2.0 g L^−1^; K_2_HPO_4_, 1.0 g L^−1^; NaH_2_PO_4_  × H_2_O, 0.4 g L^−1^; MgSO_4_  × 7H_2_O, 0.5 g L^−1^; yeast extract, 2.0 g L^−1^; pH 6.5) with 25 mycelium discs (Ø 0.5 cm) from 7-day old MA culture; (ii) incubation at room temperature (22 ± 2°C), on a rotary shaker (100 rpm), for 7 days; (iii) washing of the resultant biomass (3 times) with sterile distilled water (dH_2_O); and (iv) biomass homogenization with 100.0 mL of sterile dH_2_O in a laboratory blender.

Selenium was tested in the form of sodium selenite (Na_2_SeO_3_) at concentrations of 5.0 mg L^−1^, 10.0 mg L^−1^, 20.0 mg L^−1^, 50.0 mg L^−1^, 100.0 mg L^−1^, 500.0 mg L^−1^, 1000.0 mg L^−1^, 2500.0 mg L^−1^, 5000.0 mg L^−1^, and 10000.0 mg L^−1^ added to a sterilized modified synthetic medium which was optimum for biomass production (with glucose in the concentration of 65.0 g L^−1^ and peptone as nitrogen source in the concentration of 2.0 g L^−1^). The minimum inhibitory Se concentration (MIC) was determined by mushroom cultivation in a microtitration plate containing 90.0 *μ*L of Se-amended modified synthetic medium and 10.0 *μ*L of homogenized inoculum, at 25°C for 21 days. Medium without Se was used as the control. Three replicates for each Se concentration were made.

### 2.2. Morphophysiological and Ultrastructural Analyses

Hyphal morphology and ultrastructure, biomass production, and the mycelium Se absorption ability were assessed by cultivating* P. ostreatus* in 100 mL flasks containing 20.0 mL of modified synthetic medium enriched with Se at concentrations which did not completely inhibit growth, at room temperature, on a rotary shaker for 9 days. Inoculum of 1.5 mL was used per flask.

The morphological examinations were done by observing mycelium immersed in glycerin or stained with fuchsine acid on slides using a light microscope (Carl Zeiss Axio Imager 2).

For ultrastructure analysis, the mycelium was fixed overnight at 4°C in 2.5% glutaraldehyde in 0.1 M phosphate buffer, postfixed for 1 h in the same buffer containing 1.0% osmium tetroxide, and dehydrated and embedded in Araldite resin. Ultrathin sections were cut with a UC6 ultramicrotome (Leica Microsystem, Germany), routinely stained with uranyl acetate and lead citrate, and observed under 80 kV in a Philips CM12 transmission electron microscope (Philips/FEI, The Netherlands).

After 9 days of cultivation, mycelium biomass was filtered, washed three times with dH_2_O (50.0 mL) on a magnetic stirrer at 30°C for 3 min to remove the remaining Se on cell walls, dried at 50°C to constant weight, and measured. Mycelium biomass is presented in g dry weight L^−1^ of medium.

The concentration of absorbed Se was determined by hydride generation atomic absorption spectrophotometer (HG-AAS) Model SP190 (Pye Unicam, England). Samples were prepared by digestion of 0.1 g of dried biomass with 10.0 mL of conc. HNO_3_ and 3.0 mL of conc. HCl and by further dilution with Milli-Q water to a final volume of 20.0 mL. A standard curve was created using solutions containing Se at concentrations of 0.0 *μ*g L^−1^, 10.0 *μ*g L^−1^, 25.0 *μ*g L^−1^, and 50.0 *μ*g L^−1^. Se data are presented as *μ*g of absorbed Se per g of dried biomass and as percentage of absorbed Se from the original incubation medium. Three replicates for each Se concentration were made.

The mycelium growth rate was determined by measuring the daily change of colony diameter during mushroom cultivation in Petri dishes (Ø 90 mm), with Se-enriched agarized modified synthetic medium, at 25°C for 20 days.

### 2.3. Statistical Analysis

Data are expressed as the mean ± standard error of measurements from triplicate experiments. One-way analysis of variance (ANOVA) was used to test the significance of differences among the absorbed Se concentrations, using STATISTICA software, version 5.0 (StatSoft, Inc.). *P* values less than 0.01 were considered significant.

## 3. Results and Discussion

### 3.1. The Effect of Selenium Concentrations on Mycelium Growth and Absorption Capacity

Mycelium growth was good in media enriched with Se at concentrations of 5.0 mg L^−1^, 10.0 mg L^−1^, and 20.0 mg L^−1^ and growth intensity appeared the same as in the control medium. Se concentrations of 50.0 mg L^−1^ and 100.0 mg L^−1^ were less suitable for growth, and mycelium density was lower; 500.0 mg L^−1^ of Se strongly inhibited growth, while higher Se concentrations stopped growth completely. The MIC of Se was determined to be 1000.0 mg L^−1^.

Mycelium growth rate was defined by change in colony diameter on the agarized synthetic media enriched with Se concentrations ranging from 0.0 mg L^−1^ to 500.0 mg L^−1^. Colony diameter achieved its maximum (Ø 90 mm) on day 13 of cultivation in the control as well as in media with Se at concentrations of 5.0 mg L^−1^, 10.0 mg L^−1^, and 20.0 mg L^−1^, which means that the growth dynamic was almost the same at these concentrations. Growth rate in the control increased with cultivation time, from 2.0 mm day^−1^ during the first five days to 5.0 mm day^−1^ during the last four days, and growth rates were similar in the presence of 5.0 to 20.0 mg L^−1^ Se. At Se concentrations of 50.0 mg L^−1^ and 100.0 mg L^−1^, mycelium growth was slow, with rates varying from 1.5 mm day^−1^ at the beginning of cultivation to 3.0 mm day^−1^ during the last 11 days, and the maximum colony diameter was reached on day 20 of incubation. Although the Se MIC was 1000.0 mg L^−1^, mycelium growth on agarized medium enriched with Se at 500.0 mg L^−1^ was absent even after 20 days of cultivation. Others have also found that the MIC depends on the type of cultivation [23].

**Figure 1:**
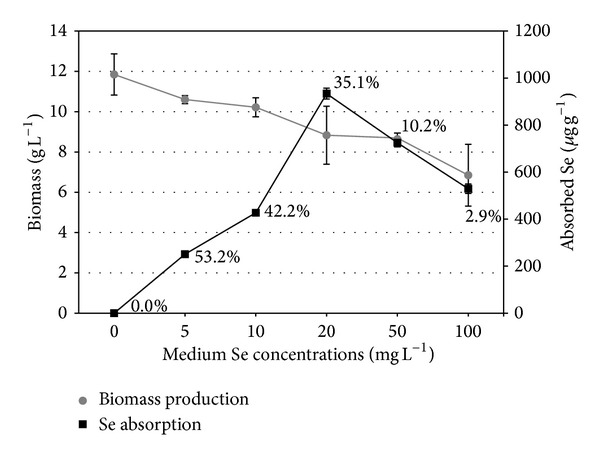
Biomass production and Se absorption by* Pleurotus ostreatus* depending on Se amount in the medium. (Data represent mean values of three samples. Variations are given as standard errors.)

Biomass production decreased as Se concentration increased. Thus, biomass decreased from 11.8 ± 1.0 g L^−1^ in the control to 6.8 ± 1.5 g L^−1^ at an Se concentration of 100.0 mg L^−1^, while production was completely inhibited at a concentration of 500.0 mg L^−1^ (Figure 1). Although colony diameters were almost identical in the control and in media enriched with the lowest three Se concentrations, biomass yield was decreased at all Se concentrations in comparison with the control (*P* < 0.01).

In the control samples,* P. ostreatus* mycelium contained no Se. However, in the Se-enriched media, Se concentrations in the mycelium ranged from 251.2 *μ*g g^−1^ (at an Se concentration of 5.0 mg L^−1^) to 938.9 *μ*g g^−1^ (at an Se concentration of 20.0 mg L^−1^) which was the maximum amount absorbed (Figure 1). At higher Se concentrations in the medium, absorption levels decreased, though concentrations in the mycelium were higher than those obtained at Se concentrations of 5.0 mg L^−1^ and 10.0 mg L^−1^ (*P* < 0.01).

Selenium content in the biomass, that is, mycelium ability for Se absorption, was not correlated positively with its concentration in the medium (Figure 1). The optimum Se concentration in the medium for the best biomass production as well as Se absorption needs further study of the range between 15.0 and 20.0 mg L^−1^, where biomass production would be approximately 9.0 g L^−1^ and Se absorbed at about 800.0 *μ*g g^−1^. This should be Se concentration range where the maximum amount of Se extracted from medium is expected. Although reduction of fungal biomass is evident with increase of Se concentration, considerable fungal growth occurred at Se concentrations up to 100 mg L^−1^ which were capable of significant Se uptake, and the range of the optimal ratio biomass/Se concentration for Se absorption is illustrated in Figure 1.

Se taken up from the medium and incorporated into the mycelium biomass, presented as percentage of total Se added to the original medium, showed a decrease with increasing medium Se concentration. The highest percentage of absorbed Se (53.25%) was found at a medium Se concentration of 5.0 mg L^−1^. Medium Se concentrations of 10.0 mg L^−1^ and 20.0 mg L^−1^ were also suitable for its absorption (43.58% and 41.31%, resp.), while the percentages significantly declined after cultivation in media enriched with 50.0 mg L^−1^ and 100.0 mg L^−1^ Se (11.17% and 3.60%, resp.).

Numerous studies have demonstrated that various species and even strains, depending on their developmental stage, substrate, and Se form and concentration, have different abilities to absorb and accumulate Se [16, 17]. Thus, these authors reported that the Se content in* Lentinula edodes* mycelium and* Saccharomyces cerevisiae *cells, cultivated in a medium enriched with Se at 20.0 mg L^−1^, was 748.0 *μ*g g^−1^ and 1825.0 *μ*g g^−1^, respectively. Contrary to these species which have significant Se uptake ability,* Ganoderma lucidum* had a low potential for Se incorporation in fruiting bodies, only 29% of the medium Se concentration [24]. According to Soković and Van Griensven [23],* P. ostreatus* HAI 592 could be considered as a hyperaccumulator because its Se absorption capacity is more than 100.0 mg per kg of dry matter which is at least about 100 times greater than the values to be expected in nonaccumulating species on the same substrate.

Selenium bioavailability also depends on its chemical forms and concentration present in the substrate as well as substrate characteristics (composition, type, temperature, pH, water salinity, and oxygen concentration) [25, 26]. A few Se forms can be found in nature: (i) elemental insoluble and less bioavailable Se (Se^0^), (ii) volatile selenide (H_2_Se) and metal selenides (metal-Se) which pass into the atmosphere through bioactivity, (iii) less bioavailable selenites (SeO_3_^2−^), (iv) mobile selenates (SeO_4_^2−^), and (v) organic Se compounds [2]. Despite the fact that Na_2_SeO_3_ is a commercially available form of Se [27], Letavayová et al. [28] showed that selenites generally are much more toxic than organic Se forms due to the generation of reactive oxygen species.

**Figure 2:**
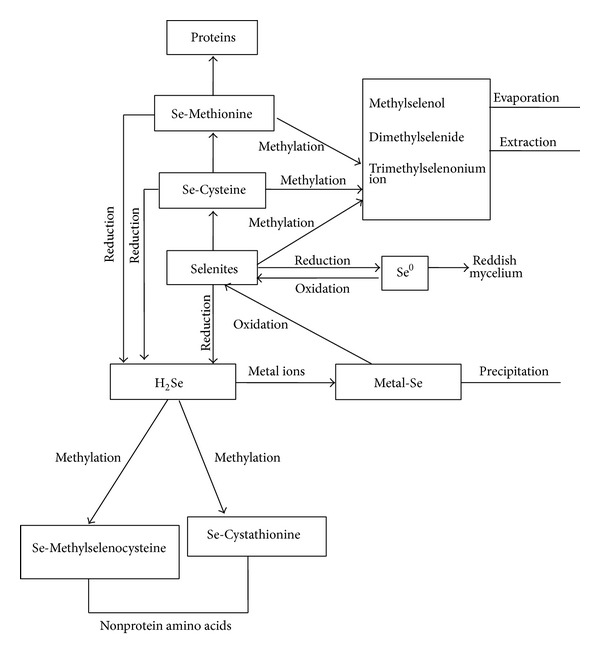
Se metabolism pathways (scheme).

Although it is known that absorbed Se is incorporated into selenocysteine, selenomethionine, Se-methylselenocysteine, and several unidentified selenocompounds [29], mechanisms of its uptake, translocation, accumulation, and metabolism depend on the species [30]. According to Ip [27] and Lenz and Lens [2], water soluble selenites and selenates could be introduced into two metabolic pathways: (i) methylation directly or indirectly (through incorporation into selenomethionine) to methylselenol, dimethyl selenide, and trimethylselenonium ion which are vaporized or excreted and (ii) reduction to insoluble Se^0^, which could be either reoxidized to selenites, rarely selenates, or reduced to H_2_Se. H_2_Se, a precursor for selenoprotein synthesis, in the reaction with metal cations forms metal-Se that further either precipitates or oxidizes to selenites (Figure 2).

Selenium forms and concentrations also act differently on the development and metabolism of various mushrooms species. Thus, Zhao et al. [24] showed that* S. cerevisiae* accumulated Se to concentrations ranging between 1200.0 *μ*g g^−1^ and 1400.0 *μ*g g^−1^ dry weight during the exponential phase of growth in Na_2_SeO_3_-enriched medium, which contained 30.0 *μ*g mL^−1^ Se, and that higher Se amounts caused strong growth inhibition. Similar results were obtained by Malinowska et al. [31], who noted a dramatic reduction of* Hericium erinaceum* mycelium yield during cultivation in a medium with Se at 100.0 mg L^−1^, while a concentration of 25.0 mg L^−1^ had no significant effect on growth. The findings are in accordance with those presented here for* P. ostreatus* HAI 592.

### 3.2. The Effect of the Selenium Concentrations on Mycelium Morphological and Ultrastructural Features

White and dense mycelium was obtained after 9 days of* P. ostreatus* cultivation in the control medium, and no morphological differences were observed between this mycelium and mycelia obtained in media enriched with Se at concentrations of 5.0 mg L^−1^, 10.0 mg L^−1^, 20.0 mg L^−1^, and 50.0 mg L^−1^. However, higher Se concentrations (100.0 mg L^−1^ and 500.0 mg L^−1^) caused the production of a brick-red colour in the mycelium and cultivation medium.

**Figure 3:**
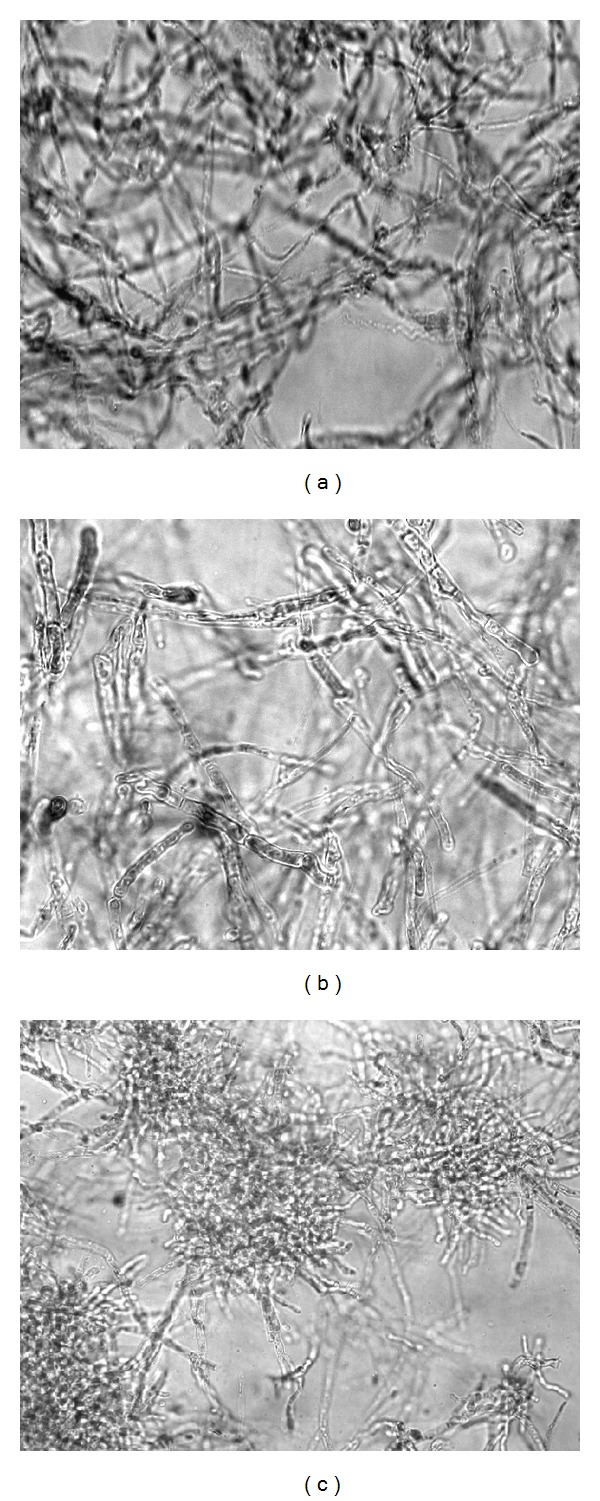
Hyphal morphology depending on Se concentration in the medium. (a) Control, (b) 100.0 mg L^−1^, and (c) 500.0 mg L^−1^ (×630).

In the control medium, hyphae were typical for* P. ostreatus*, thin-walled, hyaline, with diameter in a range from 2.00 *μ*m to 5.94 *μ*m, branched, anastomosed, and with clamp-connections (Figure 3(a)). Contrary to this, in the medium enriched with Se at 100.0 mg L^−1^, hyphal density was lower, their diameter varied between 3.24 *μ*m and 10.50 *μ*m, the cell wall was thick with more expressed extracellular matrix, clamp-connections were not noticeable, septa were abundant, and branch frequency was lower (Figure 3(b)). At the highest Se concentration where growth was observed (500.0 mg L^−1^), hyphal density was the lowest with the occurrence of characteristic hyphal coils. Hyphae were noticeably shorter and frequently septed, with diameters from 4.30 *μ*m to 7.70 *μ*m, with more intensive extracellular matrix, and without clamp-connections (Figure 3(c)). Although hyphal width was up to 10.50 *μ*m at an Se concentration of 100.0 mg L^−1^, the frequency of thick hyphae (about 7.00 *μ*m) was higher at an Se concentration of 500.0 mg L^−1^. These Se concentrations affected the density and morphology of hyphae, but the most important effect was the absence of clamp-connections.

**Figure 4:**
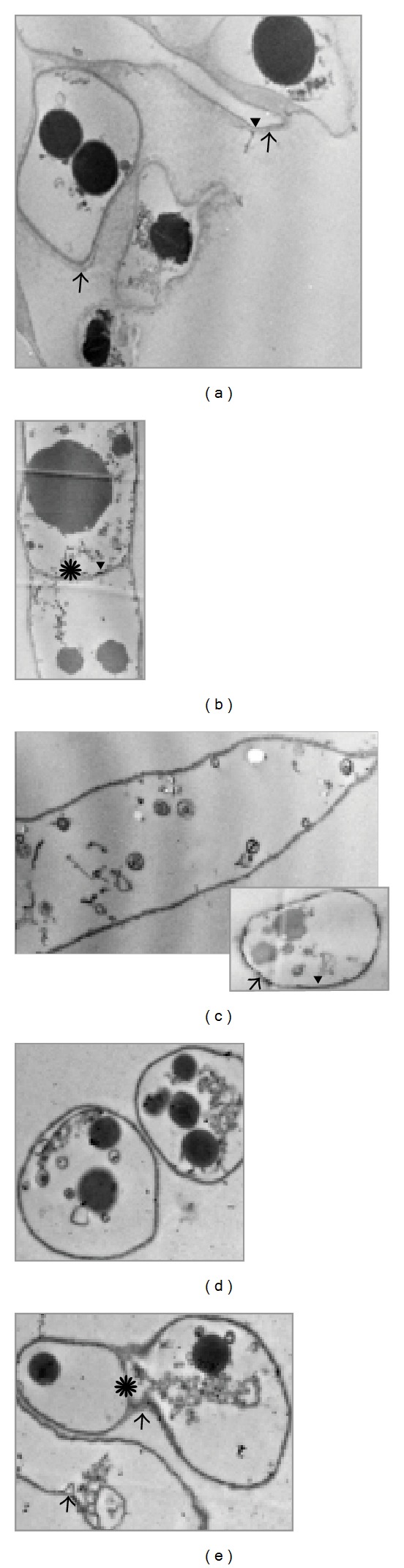
Ultrastructural features of* Pleurotus ostreatus* hyphae depending on Se concentration in the medium. (a) Control; (b) and (c) 100.0 mg L^−1^; (d) and (e) 500.0 mg L^−1^ (×15000). →: cell wall; ▼: cell membrane; *: septa.

The ultrastructural analysis showed, in comparison to the control (Figure 4(a)), that absorbed Se accumulated mainly in the fungal cell membrane and in the vacuoles, while minor changes were detected in the cell wall (Figures 4(b)–4(e)). Contrary to the cell membrane of samples cultivated in 500.0 mg L^−1^ Se-enriched medium which was completely dark, an Se concentration of 100.0 mg L^−1^ caused partial membrane darkness. Darkness of the cell and vacuolar membranes was probably the result of Se incorporation, because the main role of Se is known to be as an antioxidant agent which protects cells from lipid peroxidation caused by metal ions that accumulate in the cytosol of* P. ostreatus* [32]. Contrary to the cell wall and cell membrane, which were apparently retained intact, Figure 3 demonstrated a reduction of cellular contents positively correlated with Se concentration. Electron-dense spots, visible in both the control and Se-enriched samples, could be proteinaceous bodies as lipid bodies would be extracted during preparation for TEM. In the presence of Se, the number of these bodies increased, and changes in their shape, colour, and size were slight (Figures 4(b)–4(e)), which excludes them as candidates for Se deposition. It was also shown that the reduction of the ionic Se and production of amorphous Se^0^ are really occurring round the bodies. According to Serafin Muñoz et al. [33], chitin-containing polysaccharide structures from* P. ostreatus* cell wall as well as cytosol compounds bind Se. Therefore, binding of Se to the cell membrane and compartmentalization in vacuoles may be essential mechanisms for its detoxification.

Previous studies also showed that higher selenite concentrations caused firstly Se accumulation in* P. ostreatus *mycelium, and during the suppression growth phase, selenite was reduced to amorphous Se^0^, and this gave the mycelium and medium a reddish colour [26, 34–36]. However, besides this detoxification mechanism, Brown and Shrift [37] and Zhao et al. [24] emphasized that Se accumulators can incorporate Se from inorganic forms in Se-methylselenocysteine and Se-cystathionine, two nonprotein selenoaminoacids that are generated during the process of H_2_Se methylation. Thus, a good Se accumulator,* S. cerevisiae*, in the presence of Se excess contains selenomethionine, selenocysteine, Se-methylselenocysteine, and selenomethionine [27]. Šlejkovec et al. [18] and Díaz Huerta et al. [19] also identified selenocystathionine in supernatantsobtained from powdered fruiting bodies of* M. procera*,* L. luscina*,* B. edulis*, and* B. luridus*, and Falandysz (2008) found Se-methylselenocysteine in* L. edodes* and* A. bisporus*.

Extensive cytoplasmic vacuolization, increase of cell membrane leakage, and abundant small cytoplasmic vesicles containing electron-dense granules were also noted in* S. cerevisiae* after growth in 5.0 *μ*mol L^−1^ selenite-enriched medium [38]. However, in the presence of Se-methylselenocysteine at 10 *μ*mol L^−1^, there was no change in cell ultrastructure compared with nontreated cells.

Regarding effects of Se at the molecular level, Lenz and Lens [2] reported cell growth inhibition, decrease of DNA synthesis, DNA single-strand breaks, and cell death caused by necrosis or acute lysis as consequences of high selenite amounts. DNA double-strand breaks and cell death were also associated with the toxic and mutagenic effects of Na_2_SeO_3_ on* S. cerevisiae *[28, 39]. However, in contrast to selenites, Ip [27] and Letavayová et al. [28] observed that Se-methylselenocysteine inhibited growth rate, and DNA synthesis only modestly caused no break of DNA strands and induced cell death predominantly by apoptosis. According to these authors, activity mechanisms of selenite and Se-methylselenocysteine were also different: selenite inhibited cell growth by some nonspecific genotoxic effect, and methylated forms of Se decreased growth rate by prolongation of delay in the S phase.

It is still unclear how some species resist high Se concentrations in their environment. Gharieb and Gadd [40] proposed the main Se resistance and detoxification strategies in fungi to be the following: (i) prevention or reduction of Se entry into the cell, (ii) Se accumulation in the vacuoles, and (iii) reduction of selenite to Se^0^ or its methylation to volatile organic less-toxic forms.

## 4. Conclusion 

With rising awareness about health protection, particular attention is now given to effective Se emission control as well as to its detoxification using economically feasible and environmentally friendly procedures. The majority of filamentous fungi and yeast could be used successfully as soil bioremediators due to their (i) high Se absorption capacity; (ii) significant biosorption capability of chitin, its derivatives, as well as exopolymers (to form capsules or slime layers) based on the presence of sites for Se binding [36]; and (iii) enormous amounts of fungal biomass produced in various industrial fermentations. Although mycoremediation technology is not completely known, the results of the study presented here imply that* P. ostreatus* possesses a remarkable capacity for Se absorption and accumulation.
